# Supplementary material for: The glycosphingolipid MacCer promotes synaptic bouton formation in Drosophila by interacting with Wnt
Source: eLife. 2018 Oct 25;7:e38183. doi: 10.7554/eLife.38183 (PMC6202054; doi:10.7554/eLife.38183)
Supplement: Supplementary File 1. [file elife-38183-supp1.docx]

**Supplementary file 1.** Genetic screen for NMJ morphology by manipulating selected genes involved in synthesis and turnover of membrane lipids.

† NMJs with reduced bouton number; # NMJs with satellite boutons.

| **Function** | **Gene** | **Allele; phenotype** | **Ref.** |
| --- | --- | --- | --- |
| **Sphingolipid synthesis and turnover** | | | |
| Ceramide synthesis and turnover | *lace*  (*SPT2*) | *2/K05305; †* | (Fyrst et al., 2004) |
|  |  | *K05305/Df(2L)Exel7063; †* |  |
|  | *schlank*  (*CerS*) | *G0061; †* | (Bauer et al., 2009) |
|  |  | *G0489; †* |  |
|  | *Des1* (sphingolipid delta-4 desaturase) | *4/Df(2L)GpdhA* | (Basu and Li, 1998) |
|  |  | *1/KG00418* |  |
|  |  | *Tub>RNAi P{HMS00518}attP2* |  |
|  | *Sk2* (*Sphingosine kinase2*) | *KG05894/Df(3L)BSC671;* *#* | (Yonamine et al., 2011) |
|  |  | *Tub>RNAiP{GL00034}attP2;* *#* |  |
|  | *CerK*  (*Ceramide kinase*) | *MI04171/Df(3R)BSC177* |  |
|  |  | *Tub>RNAiP{HMS03025}attP2* |  |
|  |  | *Elav>RNAiP{HMS03025}attP2* |  |
|  | *CDase*  (*Ceramidase*) | *MB03531/Df(3R)BSC503* |  |
|  |  | *Elav>RNAiP{GL00273}attP2* |  |
|  | *Bwa* (*Brainwash*) | *e02081/Df(2L)BSC258* |  |
|  |  | *KG04628/Df(2L)BSC258; #* |  |
|  |  | *Tub>RNAiP{JF03345}attP2* |  |
|  |  | *Elav>RNAiP{JF03345}attP2* |  |
|  |  | *Elav>RNAiP{HMS03026}attP2* |  |
| PE-Cer synthesis | *CPES* | *nSyb>RNAiP{HM05273}attP2* |  |
|  | *SMSr* | *EY0609;* # |  |
|  |  | *nSyb>EY06092; #* |  |
|  |  | *PL00377* |  |
|  |  | *PL00377/Df* *(3L)Exel8104;* # |  |
|  | *CG11426* | *d05846/Df(3L)BSC420 ; #* |  |
| PE-Cer catabolism | *CG3376* | *Act>RNAiP{HMS03021}attP2; #* |  |
|  |  | *NP5300* |  |
|  | *CG12034* | *EY00448* |  |
|  |  | *Elav>RNAiP{HMS03020}attP2* |  |
|  |  | *Tub>RNAiP{HMS03020}attP2* |  |
|  | *CG32052* | *MB02409* |  |
|  |  | *Elav>RNAiP{HMS03024}attP2* |  |
| GSL synthesis | *GlcT-1* | *G5974* |  |
|  |  | *MI06082; #* |  |
|  | *egh* | *7; †* | (Wandall et al., 2003; Wandall et al., 2005) |
|  |  | *62d18; †* |  |
|  |  | *7/62d18; †* |  |
|  |  | *EP804; †* |  |
|  |  | *EY03917; †* |  |
|  |  | *Tub>EY03917; #* |  |
|  |  | *Tub>UAS-Egh; #* |  |
|  | *brn* | *fs107; #* |  |
|  |  | *1.6P6; #* |  |
|  |  | *nSyb>RNAiP{HMC04074}attP2 ; #* |  |
|  |  | *Tub>UAS-Brn; †* |  |
|  | *GalNAc-TA* | *4.1* | (Chen et al., 2007) |
|  |  | *Act>UAS-GalNAc-TA1.2; †* |  |
|  |  | *nSyb>UAS-GalNAc-TA* |  |
|  |  | *C57>UAS-GalNAc-TA* |  |
|  | *GalNAc-TB* | *nSyb>UAS-GalNAc-TB* |  |
|  | *4GT1* | *EY00269* |  |
|  |  | *EY00269/Df(2L)Exel7014* |  |
|  |  | *Tub>EY00269; #* |  |
|  |  | *Elav>EY00269* |  |
| GSL catabolism  (GlcCeramidases) | *CG33090* | *MB04916/Df(2L)FDD-0042597; #* |  |
|  |  | *5-HA-2418* |  |
|  |  | *Tub>RNAiP{HMS01576}attP2; #* |  |
|  | *CG31414* | *MB03039/Df(3R)Exel6195* |  |
|  | *CG31148* | *MB02296/Df(3R)Exel6195* |  |
| **Phospholipid synthesis and turnover** | | | |
| Phosphatidylethanolamine (PE) synthesis | *eas* (EK) | *alaE13* | (Pascual et al., 2005) |
|  |  | *KG01772* |  |
|  | *Pect* (PEth cytidylyltransferase) | *EY20477; #* |  |
|  |  | *nSyb>EY20477; #* |  |
| Phosphatidylcholine (PC) synthesis | *CCT1* | *16919/Df(3L)ED4256* |  |
|  | *CCT2* | *MB04086/Df(3L)ED4256* |  |
|  | *CG2201* | *Tub>*  *RNAiP{HMC04945}attP40* |  |
| Phosphatidylinositol (PI) synthesis | *CdsA* | *EY08412/Df(3L)BSC795* |  |
|  |  | *GS5198/Df(3L)BSC795* |  |
|  |  | *Elav>RNAi P{JF02912}attP2* |  |
|  | *Pis* | *Elav>UAS-Pis* |  |
| Phosphatidylcacid (PA) synthesis | *DGK* | *f03609* |  |
|  |  | *f03609/Df(2R)BSC265* |  |
|  |  | *MB10383* |  |
|  | *PLD* (*CG12110*) | *KG02714/Df(2R)ED1552* |  |
|  |  | *MB06221/Df(2R)ED1552* |  |
|  | *CG31140* | *MB01635/Df(3R)Exel6196* |  |
|  | *CG4729* | *EY13891/Df(3L)BSC555* |  |
|  |  | *f05465/Df(3L)BSC555* |  |
| Phospholipid lipase A2 (PLA2) | *PCID2* | *G18683/Df(3L) BSC577* |  |
|  | *iPLA-VIA* | *EY05103* |  |
|  |  | *Elav>RNAiP{HMS01544}attP2* |  |
| Lyso-Phospholipid acyltransferase (LPLAT) | *oys* | *NP5218/CB-5518-3* |  |
|  | *nes* | *f07294/Df(3L)ED4789* |  |
|  |  | *EY22898/Df(3L)ED4789* |  |
|  | *frj* | *EY06644/Df(2L)BSC185* |  |
| **Phospholipid regulation** | | | |
| sREBP processing | *sREBP/*  *HLH106* | *1/Df(3L)kto2* |  |
|  |  | *Tub>UAS-sREBP* |  |
|  |  | *Tub>RNAiP{JF01997}attP2* |  |
|  |  | *Tub>RNAiP{HMS00080}attP2; #* |  |
|  | *SCAP* | *EY06708/Df(2R)ED1484; #* |  |
|  |  | *MI00063/Df(2R)ED1484* |  |
|  |  | *Tub>UAS-SCAP.Y319C; #* |  |
|  |  | *Tub>RNAiP{JF01139}attP2 ; #* |  |
| Cardiolipin regulation | *taz* | *KG02529/Df(2R)vg-C* |  |
|  |  | *Tub>RNAiP{JF01564}attP2; #* |  |
|  |  | *Elav>RNAiP{JF01564}attP2* |  |
| **Fatty acid (FA) synthesis and turnover** | | | |
| FA synthesis | *dACC* | *Elav> RNAiP{HM04027}attP2; †* |  |
|  | *FASN* (Fatty acid synthase) | *KG03696/Df(2L)BSC162* |  |
|  |  | *5-HA-2771/Df(2L)BSC162* |  |
|  |  | *NP0872/KG03696* |  |
|  |  | *Tub>RNAiP{HM05141}attP2* |  |
|  |  | *Elav>RNAiP{HM05141}attP2* |  |
|  | *beg* | *EP3444/Df(3L)Exel7253* |  |
| FA elongation | *blp* (noa) | *NP0762* |  |
|  |  | *EY05228/Df(3L)BSC561; #* |  |
|  |  | *GS13035/NP0762* |  |
|  |  | *Elav>RNAiP{GL00227}attP2* |  |
|  | *CG2781* | *MI01455; #* |  |
|  |  | *MI01455/Df(3R)BSC196; #* |  |
|  | *Sc2* | *1/05634* |  |
|  |  | *05634/EY02236* |  |
|  |  | *1/EY02236* |  |
|  |  | *EY02236/Df(3L)Exel6098* |  |
|  |  | *Elav>EY02236; #* |  |
|  | *CG12171* | *F04657/LL07540* |  |
|  | *CG31549* | *EP3625* |  |
|  | *CG31548* | *LL01031* |  |
|  |  | *LL03771* |  |
| FA desaturation | *Desat1* | *10A* | (Kohler et al., 2009) |
|  |  | *119A/EY07679; #* |  |
|  |  | *11A/EY07679; #* |  |
|  |  | *EY07679; #* |  |
|  |  | *Tub>RNAiP{GL00428}attP2; #* |  |
|  |  | *Tub>RNAiP{HMS01654}attP40* |  |
|  | *CG9743* | *GS9520* |  |
|  |  | *GS9520/Df(3R)Exel6214;† #* |  |
|  |  | *Tub>RNAiP{HMS01563}attP2; #* |  |
|  |  | *Elav>RNAiP{HMS01563}attP2; #* |  |
| **FA binding and activation** | | | |
| Acyl-CoA synthetase (ACS) | *CG3961* | *LL01135/Df(3L)BSC776* |  |
|  |  | *NP3165/Df(3L)BSC776* |  |
|  |  | *LL01135/ NP3165* |  |
|  | *CG6178* | *EP3251/Df(3R)Exel6198; #* |  |
|  |  | *LL07332/Df(3R)Exel6198* |  |
|  |  | *GS111470/Df(3R)Exel6198; #* |  |
|  |  | *GS111470/LL07332; #* |  |
|  |  | *Tub>RNAiP{HM05128}attP2* |  |
|  | *pdgy* | *BG02662* |  |
|  |  | *EY02124/Df(1)ED7265* |  |
|  | *bgm* | *1/Df(2L)ED8186* |  |
|  |  | *K09909/Df(2L)ED8186; #* |  |
|  |  | *Elav>RNAiP{JF03054}attP2* |  |
| Acyl-CoA binding protein (ACBP) | *Anox* | *GS1189; #* |  |
|  | *CG8498* | *NP7341/Df(2L)BSC228* |  |
|  | *CG5604* | *NP2404/Df(2L)Exel7046* |  |
|  | *CG8814* | *EY12940/Df(2L)BSC180* |  |
|  | *CG14232* | *KG05353* |  |
| FA binding | *FABP* | *KG06479/Df(3R)Exel8153* |  |
|  |  | *Tub>RNAiP{HMS01163}attP2* |  |
|  | *rFABg* | *Elav>RNAiP{HM05157}attP2* |  |
|  |  | *Elav>RNAiP{HMS00265}attP2* |  |
|  | *San* | *KG04816/Df(2R)BSC703* |  |
|  |  | *Tub>RNAiP{GL00592}attP2; #* |  |
|  | *FATP* | *k10307/Df(2L)Exel7048* |  |
|  |  | *Elav>RNAiTH01670.N* |  |
